# Supplementary material for: P-TRAP: a Panicle Trait Phenotyping tool
Source: BMC Plant Biol. 2013 Aug 29;13:122. doi: 10.1186/1471-2229-13-122 (PMC3848748; doi:10.1186/1471-2229-13-122)
Supplement: Additional file 7 — P-TRAP source code. The Netbeans project that contains the source code of the application. [file 1471-2229-13-122-S7.zip › javadoc.zip!/javadoc/org-bioalg-graphprocessor/org/bioalg/graphprocessor/Vertex.html]

Vertex (GraphProcessor)


JavaScript is disabled on your browser.


- Package
- Class
- Tree
- Deprecated
- Index
- Help

*org.bioalg.graphprocessor 1.0*

- Prev Class
- Next Class

- Frames
- No Frames

- All Classes

- Summary:
- Nested |
- Field |
- Constr |
- Method

- Detail:
- Field |
- Constr |
- Method


org.bioalg.graphprocessor

## Class Vertex

- java.lang.Object
- - java.awt.geom.Point2D
  - - java.awt.Point
    - - org.bioalg.graphprocessor.Vertex

- All Implemented Interfaces:
  :   java.io.Serializable, java.lang.Cloneable

  ---

    

  ```
  public class Vertex
  extends java.awt.Point
  ```

  A graph vertex is a point with a given type.

  Author:
  :   faroq

  See Also:
  :   Serialized Form

- - ### Nested Class Summary

    Nested Classes

    | Modifier and Type | Class and Description |
    | --- | --- |
    | `static class` | `Vertex.VERTEX_TYPE` The types of the vertices. |

    - ### Nested classes/interfaces inherited from class java.awt.geom.Point2D

      `java.awt.geom.Point2D.Double, java.awt.geom.Point2D.Float`
  - ### Field Summary

    - ### Fields inherited from class java.awt.Point

      `x, y`
  - ### Constructor Summary

    Constructors

    Constructor and Description

    `Vertex()`

    `Vertex(org.w3c.dom.Element xmleleElement)`

    `Vertex(int x,
    int y,
    Vertex.VERTEX_TYPE type)`

    `Vertex(java.awt.Point point,
    Vertex.VERTEX_TYPE type)`
  - ### Method Summary

    Methods

    | Modifier and Type | Method and Description |
    | --- | --- |
    | `java.lang.String` | `getid()` |
    | `Vertex.VERTEX_TYPE` | `getType()` |
    | `boolean` | `isFixed()` |
    | `void` | `setFixed(boolean fixed)` |
    | `void` | `setType(Vertex.VERTEX_TYPE type)` |
    | `java.lang.String` | `toString()` |

    - ### Methods inherited from class java.awt.Point

      `equals, getLocation, getX, getY, move, setLocation, setLocation, setLocation, translate`
    - ### Methods inherited from class java.awt.geom.Point2D

      `clone, distance, distance, distance, distanceSq, distanceSq, distanceSq, hashCode, setLocation`
    - ### Methods inherited from class java.lang.Object

      `finalize, getClass, notify, notifyAll, wait, wait, wait`

- - ### Constructor Detail


    - #### Vertex

      ```
      public Vertex()
      ```


    - #### Vertex

      ```
      public Vertex(int x,
            int y,
            Vertex.VERTEX_TYPE type)
      ```


    - #### Vertex

      ```
      public Vertex(java.awt.Point point,
            Vertex.VERTEX_TYPE type)
      ```


    - #### Vertex

      ```
      public Vertex(org.w3c.dom.Element xmleleElement)
      ```
  - ### Method Detail


    - #### isFixed

      ```
      public boolean isFixed()
      ```


    - #### setFixed

      ```
      public void setFixed(boolean fixed)
      ```


    - #### getType

      ```
      public Vertex.VERTEX_TYPE getType()
      ```


    - #### setType

      ```
      public void setType(Vertex.VERTEX_TYPE type)
      ```


    - #### getid

      ```
      public java.lang.String getid()
      ```


    - #### toString

      ```
      public java.lang.String toString()
      ```

      **Overrides:**
      :   `toString` in class `java.awt.Point`


- Package
- Class
- Tree
- Deprecated
- Index
- Help

*org.bioalg.graphprocessor 1.0*

- Prev Class
- Next Class

- Frames
- No Frames

- All Classes

- Summary:
- Nested |
- Field |
- Constr |
- Method

- Detail:
- Field |
- Constr |
- Method
